# Peritumoral EpCAM Is an Independent Prognostic Marker after Curative Resection of HBV-Related Hepatocellular Carcinoma

**DOI:** 10.1155/2017/8495326

**Published:** 2017-05-10

**Authors:** Xiao-Meng Dai, Tao Huang, Sheng-Li Yang, Xiu-Mei Zheng, George G. Chen, Tao Zhang

**Affiliations:** ^1^Cancer Center, Union Hospital, Tongji Medical College, Huazhong University of Science and Technology, 1277 Jiefang Ave., Wuhan, Hubei 430022, China; ^2^Department of Pediatrics, Tianyou Hospital, Wuhan University of Science and Technology, Wuhan, Hubei 430064, China; ^3^Department of Surgery, Prince of Wales Hospital, The Chinese University of Hong Kong, Shatin, New Territories, Hong Kong

## Abstract

Accumulating evidence suggests that the tumor microenvironment has a profound influence on tumor initiation and progression, opening a new avenue for studying tumor biology. Nonetheless, the prognostic values of the peritumoral expression of EpCAM and CD13 remain to be elucidated in hepatocellular carcinoma (HCC) patients. In this study, the expression of EpCAM and CD13 was assessed by immunohistochemistry in peritumoral liver hepatocytes from 106 hepatitis B virus- (HBV-) related HCC patients who had undergone curative hepatectomy. The peritumoral EpCAM-positive group had a significantly worse overall survival (OS) (*p* = 0.003) and recurrence-free survival (RFS) (*p* = 0.022) compared to the negative group. Peritumoral CD13-positive patients were also associated with poor OS (*p* = 0.038), while not significantly associated with RFS. The adjusted multivariate COX proportional hazard regression analysis suggested that only the positive expression of peritumoral EpCAM precisely predicted poor OS. Being peritumoral EpCAM positive was also significantly associated with a larger tumor size, liver cirrhosis, and more frequent vascular invasion; however, no statistically significant association was observed between CD13 and any clinicopathological features. Taken together, peritumoral EpCAM and CD13 expression was associated with a poor prognosis, but EpCAM may be a better prognostic marker than CD13 in HBV-related HCC patients. In the future, peritumoral EpCAM could be a good target for adjuvant therapy after curative hepatectomy.

## 1. Introduction

Hepatocellular carcinoma (HCC) is one of the most common and aggressive human malignant tumors globally [1]. Chronic hepatitis B virus (HBV) infection is a major risk factor for HCC development, which affects approximately 250 million people worldwide [2]. Despite great improvement in the diagnosis and treatment of HCC, especially surgical and targeted therapies, the prognosis remains dismal due to metastasis or recurrence [3]. Recurrence or metastasis of HCC is mainly intrahepatic, which shows that peritumoral liver tissue may be a favorable soil for spreading hepatoma cells [4]. At the same time, a series of studies has confirmed that the tumor microenvironment has a profound influence on tumor initiation and progression, opening a new avenue for studying tumor biology [5, 6]. Even when completely curative hepatectomy has taken place, the microenvironment favorable for HCC initiation and progression still persists [7]. However, the biomarkers used to predict the prognosis of HCC patients are currently taken mainly from removed tumor tissue, and scant information is available from peritumoral tissue. Despite the extensive study of biomarkers, the results remain unsatisfactory for predicting the prognosis of HCC patients after completely curative surgical resection [8]. Therefore, it is necessary to seek optimal biomarkers for better predicting early recurrence and metastasis in peritumoral tissue.

Cancer stem cells (CSCs) (or tumor-initiating cells) are a new subpopulation that can self-renew and differentiate to produce malignant cells [9]. CSCs are considered responsible for cancer relapse and metastases owing to resistance to anticancer therapy. HCC with stem cell features has a very bad prognosis [10]. EpCAM and CD13 are some stemness-related markers in HCC [11, 12], and several studies have demonstrated that EpCAM or CD13 expression in HCC tumor tissue is associated with a poor prognosis [13, 14]. However, to the best of our knowledge, related studies about whether the expression of EpCAM or CD13 in peritumoral liver tissue has a bad prognosis in HCC have not previously been reported.

In the present study, we investigated the expression of EpCAM and CD13 by immunohistochemistry from 106 HBV-related HCC patients who had received curative hepatectomy, and we analyzed whether this expression correlated with the overall survival (OS) and recurrence-free survival (RFS).

## 2. Methods

### 2.1. Patients and Clinicopathology Information

From November 1995 to January 2013, we prospectively recruited 106 HBV-related HCC patients who underwent curative hepatectomy at the Prince of Wales Hospital, Hong Kong. The study was carried out strictly according to the reporting recommendations for tumor marker prognostic studies (REMARK) [15] and the transparent reporting of a multivariable prediction model for individual prognosis or diagnosis (TRIPOD) [16] statement. The criteria for patient inclusion have been described previously [17]. All samples and clinicopathological information were obtained with informed consent from patients or their legal representative. Curative resection was defined as the complete removal of cancer tissues with tumor-negative resection margins. Following curative resection, all liver specimens were histologically documented by two independent pathologists blinded to all patient related information. Biochemical markers, including *α*-fetoprotein (AFP), albumin, alanine aminotransferase (ALT), and bilirubin, were acquired from the patients' medical records. See detailed clinicopathological features in Table 1. The study was approved by the Joint Chinese University of Hong Kong–New Territories East Cluster Clinical Research Ethics Committee.

### 2.2. Follow-Up

All patients were followed until November 8, 2014, with a median observation time of 106.3 months. Patients were followed up by clinic visit every three months in the first year after surgery, every four months during the postoperative second year, and every six months thereafter. A contrast-enhanced abdomen CT or MRI scan was performed at least every three months during the postoperative follow-up. The death information of patients was obtained from the social security death index, medical records, or notifications from the family of the deceased.

### 2.3. Immunohistochemical Analysis and Western Blotting

We constructed a peritumoral tissue microarray (TMA) as described previously [18]. To construct the TMA slides, we used peritumoral liver tissue adjacent to the tumor within a distance of 20 mm. The expression of EpCAM or CD13 was assessed in peritumoral hepatocytes. Immunohistochemistry was carried out according to appropriate protocols, as described previously [19]. EpCAM or CD13 expression levels were semiquantitatively analyzed. The proportion score (0–3) was assigned based on the proportion of peritumoral hepatocytes within positive cytoplasmic/membranous staining, as follows: 0—staining in <1% of peritumoral hepatocytes; 1—weak staining in ≥1%; 2—moderate staining in ≥1%; and 3—strong staining in ≥1% of hepatocytes. Staining scores of 2 and 3 were defined as positive staining, whereas 0 and 1 were regarded as negative staining [20]. Western blotting was performed as previously reported [21] in 6 HCC peritumoral tissues.

### 2.4. Statistical Analysis

Analysis was performed with SPSS® version 16.0 (IBM, Armonk, NY, USA). Continuous data were exhibited as their median (range). The Pearson *χ*^2^ test or Fisher's exact test was used to analyze the correlations between immunostaining parameters and clinicopathological features. Overall survival time and recurrence-free survival time were defined, respectively, as the interval between the dates of curative hepatectomy and death or first recurrence. Data was censored at the last follow-up (November 8, 2014) for patients without death or recurrence. A Kaplan–Meier analysis was used to determine the survival and recurrence. A log-rank test was used to compare patients' survival and recurrence between subgroups; the Cox regression model was used to perform univariate and multivariate analysis. Two-tailed *p* < 0.05 was considered statistically significant for each analysis.

## 3. Results

### 3.1. Patient Demographic and Clinicopathological Characteristics

One hundred and six HBV-related HCC patients underwent curative resection at the Prince of Wales Hospital, Hong Kong, from November 1995 to January 2013. Tables 1 and 2 summarize the baseline characteristics of the 106 patients included in the study. Based on our previous study, we defined the serum AFP cutoff as 400 ng/mL [22]. The median follow-up time was 106.3 (24–215) months.

**Figure 1:**
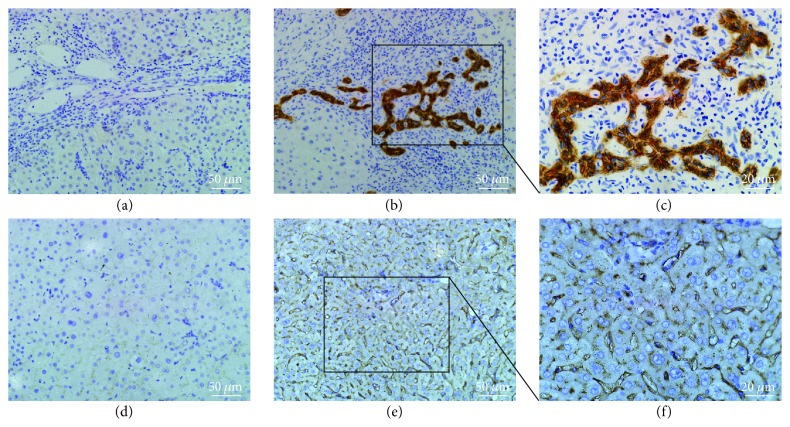
Immunohistochemical analyses of EpCAM and CD13 in peritumoral liver tissues. Negative hepatocellular cytoplasmic/membranous staining for EpCAM (a) and CD13 (d), original magnification, ×200 (scale bar, 50 *μ*m); positive staining for EpCAM and CD13, ×200 (scale bar, 50 *μ*m) (b) and (e), respectively, and ×400 (scale bar, 20 *μ*m) (c) and (f), respectively.

We analyzed the EpCAM and CD13 expression of the 106 HBV-associated HCC tumors by immunohistochemistry. The EpCAM- and CD13-positive cytoplasmic/membranous staining methodologies are shown in Figure 1. A total of 53 patients (50%) were CD13 positive, and 40 patients (38%) were EpCAM positive. To further confirm our findings, western blotting was also applied to detect the protein levels of EpCAM and CD13 in 6 HCC peritumoral tissues, 3 of them were positive immunohistochemical staining for both EpCAM and CD13, the rest were negative immunohistochemical staining for both EpCAM and CD13. As shown in Supplementary Figure S1 available online at https://doi.org/10.1155/2017/8495326, the results of western blotting were consistent with the results of immunohistochemistry. As shown in Table 2, being EpCAM positive tended to correlate with a larger tumor size (*p* = 0.040), liver cirrhosis (*p* = 0.023), and more frequent vascular invasion (*p* = 0.002). However, no statistically significant association was observed between CD13 and any clinicopathological features.

### 3.2. The Prognostic Value of Peritumoral EpCAM and CD13 Expression for HCCs in Univariate and Multivariate Analysis

**Figure 2:**
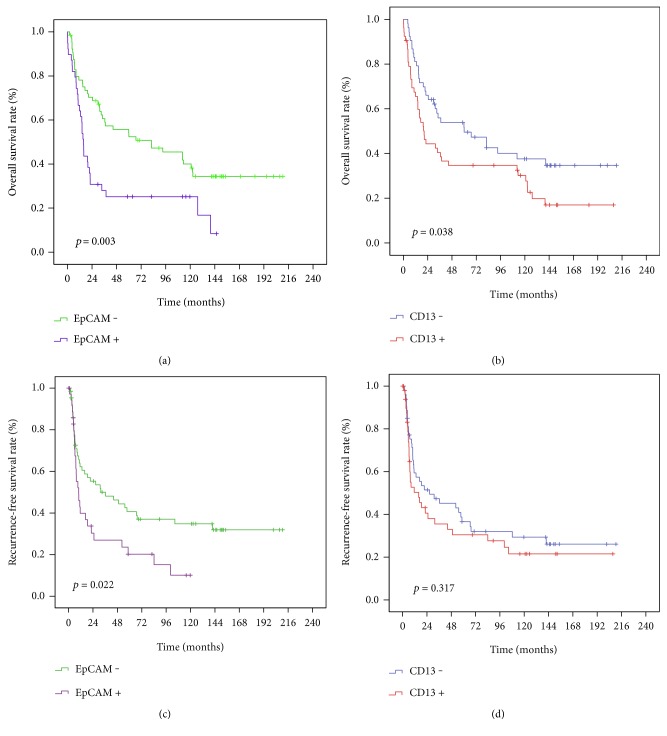
Prognostic values of the peritumoral hepatocellular expression of EpCAM and CD13. Kaplan–Meier overall survival (OS) and recurrence-free survival (RFS) analysis of different types stratified by EpCAM (a, c) and CD13 (b, d) in all 106 HBV-related HCC patients.

We performed Kaplan–Meier survival and recurrence analysis of the 106 HBV-related HCC patients. As shown in Figure 2, the peritumoral EpCAM-positive group had a significantly worse OS (*p* = 0.003) and RFS (*p* = 0.022) compared to the negative group. In addition, peritumoral CD13-positive patients were also associated with poor OS (*p* = 0.038), whereas no significant result was observed between CD13 and RFS.

Our univariate analysis revealed that an age ≥ 50, AFP ≥ 400 ng/mL, alanine aminotransferase (ALT) > 80 IU/L, multiple tumors, macroscopic vascular invasion, liver cirrhosis, and a tumor size greater than 5 cm were all statistically significant predictors of poor survival in HBV-related HCC patients (Table 3). At the same time, AFP ≥ 400 ng/mL, multiple tumors, macroscopic vascular invasion, and liver cirrhosis were also statistically associated with poor RFS (Table 4). The univariate Cox proportional HR of being peritumoral EpCAM positive versus EpCAM negative was 2.053 (1.272–3.313) (*p* = 0.003) for OS, and 1.767 (1.078–2.895) (*p* = 0.024) for RFS; the univariate Cox proportional HR of being peritumoral CD13 positive versus CD13 negative was 1.625 (1.022–2.502) (*p* = 0.040) for OS but was not statistically significant for RFS.

The multivariate Cox proportional hazards analysis was performed based on factors that were demonstrated to be significant in univariate analysis; AFP ≥ 400 ng/mL, multiple tumors, macroscopic vascular invasion, and liver cirrhosis all independently and significantly increased both the mortality and recurrence of HCC (Tables 3 and 4). In the multivariable model, the adjusted Cox proportional HR for peritumoral EpCAM-positive patients was 2.030 (1.252–3.290) (*p* = 0.004) for OS, while it was not statistically significant for RFS; at the same time, the adjusted HR for being peritumoral CD13 positive was also not statistically significant for OS. Taken together, peritumoral EpCAM may be a better prognostic marker than CD13 in HBV-related HCC patients.

## 4. Discussion

To our knowledge, this is the first study to identify peritumoral EpCAM as an independent prognostic factor for HBV-related HCC after curative resection. Patients with positive peritumoral hepatocellular EpCAM expression had a significantly increased risk of death and recurrence compared with the negative subgroup. Patients with positive CD13 expression in peritumoral tissue were only significantly associated with poor OS but were not significantly associated with RFS. The adjusted COX proportional hazard regression analysis also suggested that only the positive expression of peritumoral EpCAM precisely predicted poor OS, suggesting that peritumoral EpCAM may be a better prognostic marker than CD13 in HBV-related HCC patients. Therefore, more frequent follow-up may be required for HBV-related HCC patients with positive peritumoral EpCAM or CD13 expression (especially EpCAM positive) after curative resection, and peritumoral EpCAM could also serve as a new biomarker predicting HCC recurrence.

HCC relapse included two models: (1) a true metastasis by subclinical metastatic HCC cells' dissemination, and (2) a new neoplasm of residual liver tissue after hepatectomy caused by chronic virus infection and inflammation [23]. Increasing evidence suggests that the existence of CSCs may play a crucial role in metastases and recurrence for HCC patients after curative resection [20]. EpCAM is a type I transmembrane glycoprotein that was initially shown to be a hepatic stemness marker by Schmelzer et al. in 2006 [24]. In liver tissues, enhanced expression of EpCAM is closely associated with the proliferation of liver cells, both normal and malignant [25]. Relevant research exhibited EpCAM may facilitate HCC formation by the activation Wnt–*β*-catenin signaling [21]. Moreover, a study recently demonstrated that EpCAM expression in hepatocellular carcinoma cell lines was associated with chemoresistence, in favor of the recurrence for HCC patients [26]. CD13, also called amino peptidase N, is a super family of zinc-binding metalloproteinases, which was first reported to be a hepatic stemness marker by Haraguchi et al. in 2010 [12]. CD13 plays a role in cellular processes such as cell adhesion, angiogenesis, mitosis, invasion, antiapoptosis, and radiation resistance, which plays an important role in cancer initiation and progression [27–29]. Most recently, it was found as a marker for dormant or semiquiescent CSCs in human HCC cancer cell lines and clinical samples [30]; CD13+ HCC cells have also been exhibited to be highly chemoresistant to 5-fluorouracil and doxorubicin treatment [12]. Moreover, chronic inflammation, caused by HBV/HCV infection, also can increase stemness (EpCAM and CD13), which may be in favor of tumor recurrence [31]. Several studies have demonstrated that EpCAM or CD13 as stemness markers are associated with a poor prognosis in various types of cancer [13, 14, 32, 33]. However, scant information is known about the role of EpCAM and CD13 in peritumoral liver tissues. In the present study, we found that peritumoral EpCAM and CD13 can be poor prognosis risk factors. Being peritumoral EpCAM positive was also significantly associated with larger tumor size, liver cirrhosis, and more frequent vascular invasion. At the same time, multivariate Cox regression analysis identified AFP ≥ 400 ng/mL, multiple tumors, macroscopic vascular invasion, and liver cirrhosis as independent prognostic factors for OS and RFS. Therefore, peritumoral tissues expressing abundant EpCAM or CD13 may either provide fertile soil for the spreading of primary tumor and subclinical metastatic tumor cells or form a new neoplasm after curative hepatectomy caused by chronic virus infection and inflammation, eventually leading to recurrence and metastases.

Recently, a study demonstrated that hepatic progenitor cells (HPCs) or hepatic stem cells can form a ductular reaction (DR) and that the DR significantly correlated with necroinflammation, fibrosis, HBsAg, multiple nodules, the absence of a tumor capsule, severe microscopic vascular invasion, and early recurrence [34]. Therefore, we speculated that the positive expression of EpCAM or CD13 in peritumoral hepatocytes may show the activation of HPCs, which could further promote ductular reaction and the formation of inflammation and fibrosis. The activation of HPCs may be favorable to the occurrence of a new neoplasm and may provide fertile soil for subclinical metastatic tumor cells for HBV-related HCC after curative resection. As we have known, recurrence or metastasis is mainly intrahepatic for HBV-related HCC after curative resection, which exhibits that peritumoral microenvironment may play an important role in HCC recurrence and metastasis. Although when completely curative hepatectomy has taken place, the microenvironment favorable for HCC initiation and progression still persists. However, although we defined peritumoral tissues as liver tissues adjacent to the tumor within a distance of 20 mm which were relatively far away from tumor tissues, peritumoral tissue has also been excised after curative resection. Whether resected peritumoral tissues can effectively represent peritumoral microenvironment, future comparative study including liver tissue at a distance from the tumor mass needs to be designed. At the same time, our findings are limited to the prognosis significance of EpCAM and CD13 in liver peritumoral tissue. Further experiments are needed to reveal the related mechanisms with respect to how the abundant peritumoral EpCAM or CD13 provide fertile soil for tumor cells.

In conclusion, we first reported that the EpCAM expression was associated with a poor prognosis in liver peritumoral tissues. At the same time, our study also implied that future anticancer therapy should target not only residual tumor cells but also the soil for promoting tumor growth. Peritumoral EpCAM could be a good target for adjuvant therapy after curative hepatectomy.

## Supplementary Material

Supplementary Figure S1. The results (EpCAM and CD13 positive/negative expression) of immunohistochemical analysis were further validated by western blotting in 6 HCC peritumoral tissues.

## Figures and Tables

**Table 1:** Main demographic, biochemical, and clinical characteristics of the 106 HCC patients.

Variable	Unit	Value
Age	Years	56.3 (13–82)
Gender	Male	83 (78.3)
Albumin	g/L	38.2 (29–46)
ALT	U/L	48.6 (11–227)
Total bilirubin	g/L	11.2 (3–20)
HCC diameter	cm	5.1 (1.1–15)
AFP	ng/mL	97 (2–699,800)

Data are presented as median value (range) or absolute frequency (%); ALT: alanine aminotransferase; HCC: hepatocellular carcinoma.

**Table 2:** Correlations of EpCAM and CD13 protein expression in peritumoral tissues with clinicopathological characteristics.

Parameters	EpCAM		*p*	CD13		*p*
	Positive	Negative		Positive	Negative	
Age			0.163			0.418
<50 y	27	11		17	21	
≥50 y	39	29		36	32	
Gender			0.512			0.814
Male	53	30		42	41	
Female	13	10		11	12	
Cirrhosis			0.023^∗^			0.693
Absence	33	11		21	23	
Presence	33	29		32	30	
Tumor size			0.040^∗^			0.119
<5 cm	35	13		28	20	
≥5 cm	31	27		25	33	
AFP			0.422			0.169
<400 *μ*g/L	36	25		34	27	
≥400 *μ*g/L	30	15		19	26	
Histological grade			0.983			0.587
Well and moderate	56	34		44	46	
Poor	10	6		9	7	
Vascular invasion			0.002^∗^			0.643
Absence	57	24		42	40	
Presence	9	16		11	13	
Number of tumor lesions			0.722			0.667
Single	44	28		35	37	
Multiple	22	12		18	16	

*∗* indicates *p* < 0.05.

**Table 3:** Cox proportional hazard regression analysis of patients' overall survival.

Variables	Univariable			Multivariable		
	Hazard ratio	95% CI	*p* value	Hazard ratio	95% CI	*p* value
Gender (female versus male)	0.785	0.493–1.332	0.407	—	—	—
Age (<50 versus ≥50)	0.551	0.316–0.830	0.004^∗^	0.608	0.331–1.111	0.106
AFP (≥400 ng/mL versus <400 ng/mL)	1.608	1.124–2.300	0.009^∗^	1.657	1.149–2.387	0.007^∗^
ALT (>80 IU/L versus ≤80 IU/L)	1.473	1.016–2.135	0.041^∗^	1.299	0.952–2.113	0.541
Albumin (>35 g/L versus ≤35 g/L)	0.327	0.540–1.228	0.327	—	—	—
Bilirubin (>20 *μ*mol/L versus ≤20 *μ*mol/L)	1.380	0.747–2.551	0.304	—	—	—
Number of tumor lesions (single versus multiple)	0.438	0.296–0.648	0.000^∗^	0.482	0.297–0.783	0.003∗
Vascular invasion (absent versus present)	0.468	0.307–0.712	0.006^∗^	0.476	0.277–0.819	0.007∗
Cirrhosis (absent versus present)	0.553	0.385–0.792	0.001^∗^	0.543	0.370–0.797	0.002∗
Histological differentiation						
Moderately versus well	1.146	0.674–1.948	0.614	—	—	—
Poorly versus well	1.593	0.840–3.020	0.153	—	—	—
Greatest tumor diameter						
(≥5 cm versus <5 cm)	1.619	1.012–2.591	0.044^∗^	1.422	0.976–2.074	0.067
EpCAM (positive versus negative)	2.053	1.272–3.313	0.003^∗^	2.030	1.252–3.290	0.004^∗^
CD13 (positive versus negative)	1.625	1.022–2.502	0.040^∗^	1.469	0.908–2.378	0.117

CI: confidence interval; AFP: alpha-fetoprotein; ALT: alanine aminotransferase; *∗* indicates *p* < 0.05.

**Table 4:** Cox proportional hazard regression analysis of recurrence-free survival.

Variables	Univariable			Multivariable		
	Hazard ratio	95% CI	*p* value	Hazard ratio	95% CI	*p* value
Gender (female versus male)	0.695	0.417–1.156	0.161	—	—	—
Age (<50 versus ≥50)	0.762	0.521–1.235	0.167	—	—	—
AFP (≥400 ng/mL versus <400 ng/mL)	1.752	1.223–2.510	0.002^∗^	1.685	1.168–2.430	0.005^∗^
ALT (>80 IU/L versus ≤80 IU/L)	1.086	0.754–1.563	0.659	—	—	—
Albumin (>35 g/L versus ≤35 g/L)	0.767	0.528–1.114	0.164	—	—	—
Bilirubin (>20 *μ*mol/L versus ≤20 *μ*mol/L)	1.341	0.747–2.409	0.326	—	—	—
Number of tumor lesions (single versus multiple)	0.305	0.204–0.456	0.000^∗^	0.484	0.290–0.808	0.006^∗^
Macroscopic vascular invasion (absent versus present)	0.418	0.272–0.645	0.000^∗^	0.463	0.298–0.720	0.001^∗^
Cirrhosis (absent versus present)	0.681	0.476–0.972	0.034^∗^	0.604	0.419–0.872	0.007^∗^
Histological differentiation						
Moderately versus well	1.060	0.624–1.800	0.830	—	—	—
Poorly versus well	1.374	0.720–2.623	0.336	—	—	—
Greatest tumor diameter						
(≥5 cm versus < 5 cm)	1.204	0.854–1.697	0.290	—	—	—
EpCAM (positive versus negative)	1.767	1.078–2.895	0.024^∗^	1.622	0.986–2.669	0.057
CD13 (positive versus negative)	1.155	0.702–1.901	0.570	—	—	—

CI: confidence interval; AFP: alpha-fetoprotein; ALT: alanine aminotransferase; *∗* indicates *p* < 0.05.
