# Corrigendum to “Peritumoral EpCAM Is an Independent Prognostic Marker after Curative Resection of HBV-Related Hepatocellular Carcinoma”

**DOI:** 10.1155/2017/3765279

**Published:** 2017-09-20

**Authors:** Xiao-Meng Dai, Tao Huang, Sheng-Li Yang, Xiu-Mei Zheng, George G. Chen, Tao Zhang

**Affiliations:** ^1^Cancer Center, Union Hospital, Tongji Medical College, Huazhong University of Science and Technology, Wuhan 430022, China; ^2^Department of Pediatrics, Tianyou Hospital, Wuhan University of Science and Technology, Wuhan, Hubei 430064, China; ^3^Department of Surgery, Prince of Wales Hospital, The Chinese University of Hong Kong, Shatin, New Territories, Hong Kong

In the article titled “Peritumoral EpCAM Is an Independent Prognostic Marker after Curative Resection of HBV-Related Hepatocellular Carcinoma” [1], affiliation number one was given incorrectly. The corrected affiliation is shown above.
