# Supplementary figures and images for: Peritumoral EpCAM Is an Independent Prognostic Marker after Curative Resection of HBV-Related Hepatocellular Carcinoma
Source: Dis Markers. 2017 May 10;2017:8495326. doi: 10.1155/2017/8495326 (PMC5442434; doi:10.1155/2017/8495326)

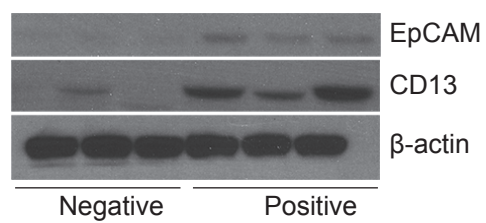

Supplement: Supplementary file 1 — Supplementary Figure S1. The results (EpCAM and CD13 positive/negative expression) of immunohistochemical analysis were further validated by western blotting in 6 HCC peritumoral tissues. [file 8495326.f1.pdf]
